# Safety and performance evaluation of the automated helical suturing system as compared with the manual suturing platform in endoscopic sleeve gastroplasty in a porcine model

**DOI:** 10.1016/j.igie.2023.10.002

**Published:** 2023-10-12

**Authors:** Steven E. Shamah

**Affiliations:** Gastroenterology and Hepatology Division, Emek Hospital, Afula, Israel

## Abstract

**Background and Aims:**

Endoscopic sleeve gastroplasty (ESG) and gastric volume reduction techniques have been shown to achieve significant weight loss and improvement in comorbid conditions. The objective of this study was to assess the safety, applicability, and usability of a novel fully automated, operator-independent endoscopic suturing system for minimally invasive treatment of obesity in pigs.

**Methods:**

Endoscopic procedures were performed in 6 domestic swine that were subjected to an internal gastric segmentation within the stomach using the EndoZip Implant (Nitinotes Ltd, Caesarea, Israel) followed by a similar endoscopic suturing system, the OverStitch ESG Implant (Apollo Endosurgery, Austin, Tex, USA) as the reference/control item. The EndoZip and OverStitch sutures were applied in each animal at 2 adjacent sites in the stomach. After each procedure, a usability questionnaire was filled out by the physician. Three animals (group 1) were monitored for a period of 7 days and 3 animals (group 2) for a period of 28 days after the procedures. On termination, segmentation sites were inspected by gross pathology examination.

**Results:**

No clinically significant abnormal clinical signs were observed during the follow-up period. Blood tests showed normal levels, and abnormal findings were observed during gross pathology examination but were not clinically significant. The histopathologic evaluation showed the tissue reaction was very similar histologically in both the ESG and automated helical suturing system samples and within expected ranges for a foreign body reaction. All 6 automated helical suturing system suture patterns were identical, demonstrating consistent tunnel creation.

**Conclusions:**

Under the conditions of this study, the automated helical suturing system was safe in this preclinical study, and further studies are required. The tissue reaction was moderate and focal and within expected ranges for a foreign body reaction (implantation of a device) in both the EndoZip and OverStitch samples.

Obesity and its attendant conditions have become major health problems worldwide. With high cost and mortality, it is the second leading cause of preventable death in the United States.^1^ Obesity is the driving factor leading to a plethora of related adverse events^2^ such as diabetes, high blood pressure, sleep apnea, stroke, depression, asthma, chronic pulmonary disease, cancer, fatty liver disease, coronary artery disease, osteoarthritis, and infertility.^3^ Although bariatric surgery is considered as the most effective method for controlling obesity,^4^ resulting in reduction of the gastric volume by 75% to 80%,^5^ less than 1% of those who qualify for bariatric surgery undergo it because of the high cost and procedure-related adverse events.^6^ Of the endoscopic options for weight loss, the most prevalent is endoscopic sleeve gastroplasty (ESG), which delivers a restrictive mechanism of weight loss with delayed gastric emptying.^7^

The automated helical suturing system, the EndoZip (Nitinotes Ltd, Caesarea, Israel), is an automatic endoscopic suturing device, and, as such, the efficacy and safety results associated with the procedure are expected to be similar to existing endoscopic suturing devices such as the OverStitch ESG (Apollo Endosurgery, Austin, Tex, USA) and primary obesity surgery endoluminal. The automated helical suturing system offers obesity treatment by an operator-friendly endoscopic procedure generating an endoluminal gastroplasty that restricts the stomach and impairs motility. It is designed to allow for the creation of multiple internal gastric segmentations in the stomach by using an endoscopic approach. In this article we describe the animal safety data and histology results of a head to head study of the automated helical suturing system, the EndoZip, versus the manual OverStitch device.

## Methods

### Study design

The study was composed of 6 female pigs weighing 70 to 110 kg. The pigs were fasted for 24 hours before anesthesia. All procedures were conducted by a single physician who is experienced in bariatric procedures in humans and pigs. Before the endoscopic suturing procedures, a marketed OverTube (Steris, Mentor, OH, USA) was placed using endoscopic visual guidance, and then the physician performed 1 suture using the EndoZip device in 1 location in the stomach, using the articulation handle and Bougie curve control handle. Then, the OverStitch procedure was performed in a different location within the stomach. Shortly after completing the 2 procedures, the physician filled in a usability questionnaire related to the EndoZip system and procedure-related parameters. Three animals were observed for a period of 7 days (group 1), and the other 3 (group 2) were monitored for 28 days after the procedures.

### Test item: the EndoZip system

**Figure 1:**
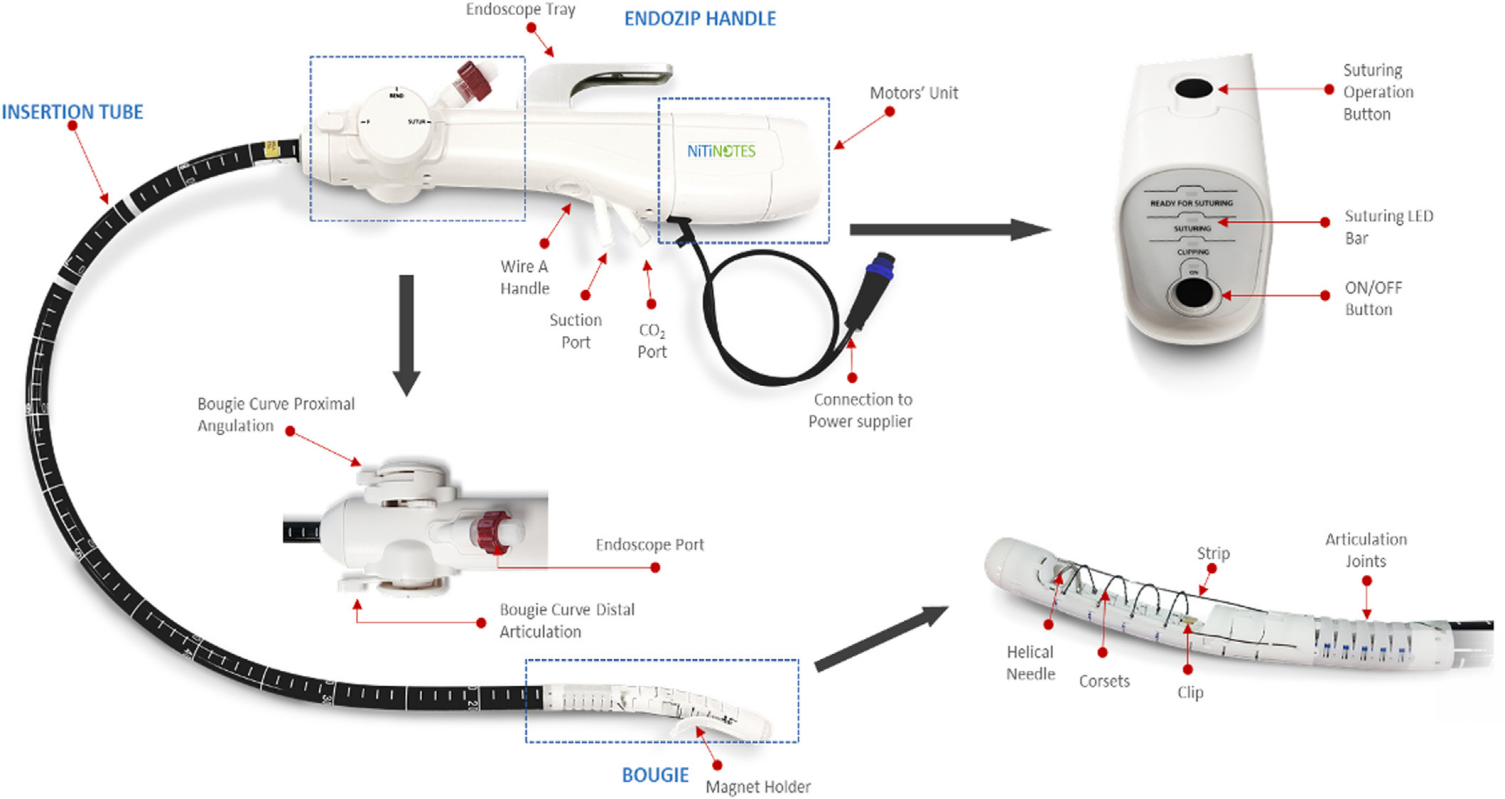
Components of the EndoZip disposable device.

The EndoZip system consists of a disposable device that controls the procedure and is composed of 3 main components (Fig. 1; Video 1, available online at www.igiejournal.org). First, the distal end of the device, termed the “bougie,” is designed to optimize the suturing mechanism. It contains a chamber that captures the tissue using a vacuum to support full-thickness sutures. In addition, the bougie includes an adjustable, flexible, cylinder-shaped element that eases device maneuvering. The second component, the insertion tube, connects between the handle and the bougie and supports irrigation and evacuation of air and CO_2_ and contains a channel for the endoscope to pass through. The third component is the handle, which aids the physician in maneuvering the device and positioning it in the desired abdominal area. In addition, it controls all suturing activity.

### EndoZip system principles of operation

The EndoZip is connected to a dedicated power supply that is plugged into the power source (standard wall outlet). Before placement, a small-diameter (up to 6 mm) endoscope is placed through the device, and the EndoZip handle is connected to a standard off-the-shelf vacuum pump. The EndoZip is inserted into the stomach through a standard endoscopic OverTube, while advancement and positioning in the stomach is visually guided by the endoscope inserted through the device's dedicated channel. The physician maneuvers the bougie to the desired location in the stomach. The system enables the approximation of the opposite stomach walls by suctioning the chosen deployment site. This vacuum draws the tissue segments into the bougie and creates a narrowing for segmentation of the stomach.

After the approximation process, the physician presses on the operation button, located on the handle, to activate the suturing action. In this process, a custom-designed needle is driven through the bougie, passing the attached suture through the approximated tissue segments, and creating the continuous plication within the stomach. Then, on a second button press, the device enables tightening and cinching of the approximated tissue segment with an integrated, dedicated clip. The system provides, to the physician, a suturing stage indication by a light-emitting diode bar located on the device handle. The suturing actions are done automatically by the system, following the physician press on the operation button, and take approximately 2 minutes to complete. The procedure is completed when the corset is released and the device is retracted from the deployment site; a visual confirmation is available using a scope.

### Data evaluation

#### General observation

Animals were observed for a total duration of either 7 days (group 1) or 28 days (group 2) after the procedures. All animals were observed for morbidity, mortality, and injury at least twice daily throughout the study period. Furthermore, food and water consumption were qualitatively observed at least once daily. Clinical and laboratory signs of injury were assessed. Body weight was monitored in the postoperative period.

#### Necropsy

All animals were subjected to necropsy and gross pathologic examination following the respective scheduled termination. Tissue samples of abnormal findings were submitted to histopathology examination. Findings were documented, recorded, and photographed.

#### Evaluation of suturing sites

**Figure 2:**
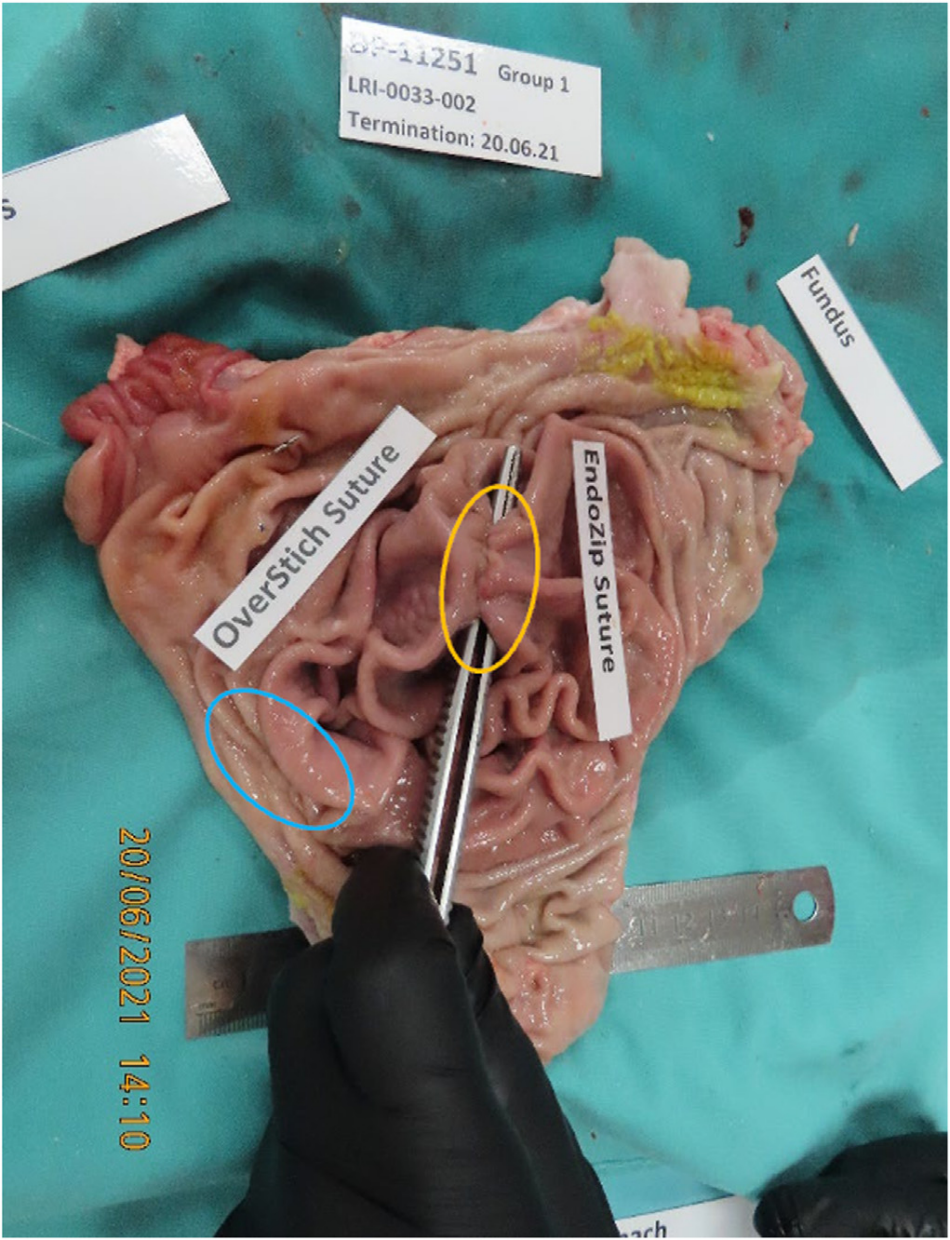
Representative picture of the EndoZip sutures (*yellow circle*) and the manual OverStitch sutures (*blue circle*).

The stomach was explanted, and the following data were collected and documented: The area, surgically treated with the test and reference items and containing the applied suturing, was visually inspected and photographed, the integrity of the sutures was inspected and documented, and the thickness of the stomach was measured using a mechanical thickness gauge. The stomach was dissected out, and a block of tissue containing the suturing sites of both procedures (ie, EndoZip and OverStitch) +2 cm was excised, macroscopically evaluated, and photographed (Fig. 2). The samples were attached to a flexible board and oriented. Suturing sites were inserted into labeled vials filled with 10% neutral buffered formalin and sent for pathology.

#### Histopathology evaluation

Tissue samples from 6 female pigs were sectioned and stained with hematoxylin and eosin. Evaluation was performed using a light microscope (BX43; Olympus; Shinjuku, Tokyo, Japan) and a digital camera (DP21; Olympus) with CellSens Entry 1.13 software (Olympus; Table 1). The tissue biologic response was evaluated using semiquantitative scoring (Table 2) of the following parameters: extent of fibrosis or fibrous capsule and inflammation, degeneration as determined by changes in tissue morphology, severity of inflammatory response and cell types, and the presence and extent of necrosis.

**Table 1:** Schematic representation of the slides (H&E, orig. mag. ×100)

Test device: automated helical suturing system			Reference device: endoscopic sleeve gastroplasty		
1	2	3	1	2	3
Stomach distal	Stomach middle	Stomach proximal	Stomach distal	Stomach middle	Stomach proximal

**Table 2:** Parameters evaluated for tissue response around the device

Response	Score				
	0	1	2	3	4
Surrounding fibrosis	0-.5 mm	Narrow band around device space .6-1.5 mm	Moderately thick band from device space 1.6-3.5 mm	Thick band from device space 3.6-5 mm	Extensive band from device space ≥6 mm
Cell type response					
Granulocytes	0	Minimal infiltration	Mild infiltration	Moderate infiltration	Severe infiltration
Mononuclear cells	0	Minimal infiltration	Mild infiltration	Moderate infiltration	Severe infiltration
Macrophages	0	Minimal infiltration	Mild infiltration	Moderate infiltration	Severe infiltration
Necrosis, extent	0	Focal around device	Locally extensive around device	Extensive around device	More than 80% of the tissue on section

### Compliance with good laboratory practice

The study described in this report was conducted in compliance with the following good laboratory practice standards: Organization for Economic Cooperation and Development Series on Principles of Good Laboratory Practice and Compliance Monitoring Number 1 and Principles on Good Laboratory Practice (as revised in 1997; ENV/MC/CHEM[98]17). These principles of good laboratory practice are accepted by the members of the Organization for Economic Cooperation and Development and the U.S. Food and Drug Administration Mutual Acceptance of Data.

### Usability and physician satisfaction

At the end of the suturing procedures, the operating physician graded various usability parameters using a semiquantitative grading of 5 grades (1-5) where grade 5 indicated the most favorable opinion and grade 1 the least favorable.

## Results

No animal died throughout the entire observation period. All animals lost minimal weight during the observation period. Group 1 lost an average of .2 kg from day 0 until day 7, whereas group 2 lost an average of .37 kg during the observation period from day 0 until day 28.

Blood and urine tests were found to be within normal ranges in all animals during the observation period. The white blood cell count was within normal range, indicating the animals did not have from systemic infection, and the red blood cell count, hemoglobin, and hematocrit were all within normal ranges, indicating the animals did not have internal bleeding.

All EndoZip suturing sights were visible. Four of 6 sutures were tight, and the wall thickness of the EndoZip suturing area was in the range of 2.6 to 4.55 mm, reaching deep into the submucosa. Three ESG sutures were observed as tight, whereas 3 were loose. The wall thickness of the suturing area was in the range of 2.7 to 7.0 mm, which also reached the submucosa. Of note, the suture pattern and thickness were identical between all 6 pigs.

The histopathology evaluation was performed by the study pathologist. Tissue reaction was very similar histologically for both the automated helical suturing system and the manual samples. The depth of the sutures and clips was very similar in all samples, which reached the deep submucosa layer. Inflammation was restricted to the areas surrounding the suturing material or clip. The clip incited a larger area of inflammation when compared with the suture because of its larger size and compression of adjacent tissue, which incited focal necrosis. In comparing the tissue reaction to the suture between the automated helical suturing system and the manual procedure, both procedures resulted in similar tissue reactions, with virtually identical scoring at 28 days as shown in Tables 3 and 4.

**Table 3:** EndoZip vs OverStitch: 7-day tissue reaction histology

Intervention	Histologic properties	Animal no.		
		DP-11251	DP-11252	DP-11253
EndoZip	Fibrosis	1	2	1
	Granulocytes	2	3	3
	Mononuclear cells	1	2	1
	Macrophages	1	1	2
	Necrosis	1	2	2
OverStitch	Fibrosis	1	2	1
	Granulocytes	1	2	1
	Mononuclear cells	1	1	1
	Macrophages	1	1	1
	Necrosis	1	1	2

Scores are .0-2.9, nonirritant; 3-8.9, slight irritant; 9-15, moderate irritant; >15, severe irritant. Negative difference is recorded as 0.

**Table 4:** EndoZip vs OverStitch: 28-day tissue reaction histology

Intervention	Histologic properties	Animal no.		
		DP-11179	DP-11180	DP-11181
EndoZip	Fibrosis	2	1	2
	Granulocytes	2	0	2
	Mononuclear cells	1	2	2
	Macrophages	3	2	3
	Necrosis	1	1	1
OverStitch	Fibrosis	1	1	3
	Granulocytes	1	2	3
	Mononuclear cells	2	1	2
	Macrophages	1	2	3
	Necrosis	1	1	1

Scores are .0-2.9, nonirritant; 3-8.9, slight irritant; 9-15, moderate irritant; >15, severe irritant. Negative difference is recorded as 0.

The usability questionnaires were assessed by the designated physician during all procedures. Scores were recorded after completion of the suturing procedures using the EndoZip system (test item). All scoring values were either 4 or 5 (except for 1 response in animal DP-11179 that was graded a 3). This indicates that the EndoZip was evaluated as easy to use.

## Discussion

A swine model was chosen because it has been used extensively as a GI model because its anatomic and physiologic characteristics are similar to humans.^8^ It is worthwhile noting that the swine model may be very challenging and represents a tougher task because the swine’s gastric capacity and surface tension are significantly greater compared with humans. Under the conditions of this study, the automated helical suturing system, the EndoZip, can be considered safe with regard to the parameters of local tolerance, evaluated by comparing the gross pathology and histopathologic findings of the suturing sites treated with the EndoZip with those treated with the Overstitch device and comparing the clinical pathology parameters and animals’ clinical signs between procedures.

The histopathology results indicated similar tissue reaction as well as similarity in the sutures and clips in terms of location within the stomach walls in both EndoZip and OverStitch procedures at both 7 and 28 days. The tissue reaction was moderate and focal in most samples and within expected ranges for a foreign body reaction (implantation of a device). In addition, no difference was found during the sutures’ visual inspection. Four of 6 automated helical sutures were observed as tight, and 3 of 6 manual ESG sutures were observed as tight.

From a usability endpoint, the physician found the automated helical suturing system easy to use as compared with ESG alternatives. However, of note, this was a single-operator study. All steps, from system connection to system retrieval, related to the EndoZip operation and procedure were evaluated as easy. In addition, each of the 6 suture patterns were identical both in form with minimal variability and depth of tissue penetrance. Reproducibility of suture depth and pattern is a serious concern with other gastric plication platforms and is believed to impact durability. The reproducibility of the automated helical suturing system, as demonstrated in the swine model, with a push button may solve this aspect and may yield a more durable sleeve gastroplasty.

In summary, the automated helical suturing system was found to be safe and easy to use in this preclinical study, and further studies are required. Future human prospective trials and prospective randomized trials comparing conservative and other endobariatric interventions are necessary to further comment on the safety and durability of the EndoZip suturing platform.

## Ethics Statement

This article does not discuss patients, and therefore no consent was needed.

## Disclosure

The following author disclosed financial relationships: S. E. Shamah: Consultant for Nitinotes Surgical and Apollo Endosurgery.
